# Supplementary figures and images for: Structural dynamics of the human Orai1 channel revealed by cryo-electron microscopy
Source: PLoS One. 2026 May 11;21(5):e0348440. doi: 10.1371/journal.pone.0348440 (PMC13160330; doi:10.1371/journal.pone.0348440)

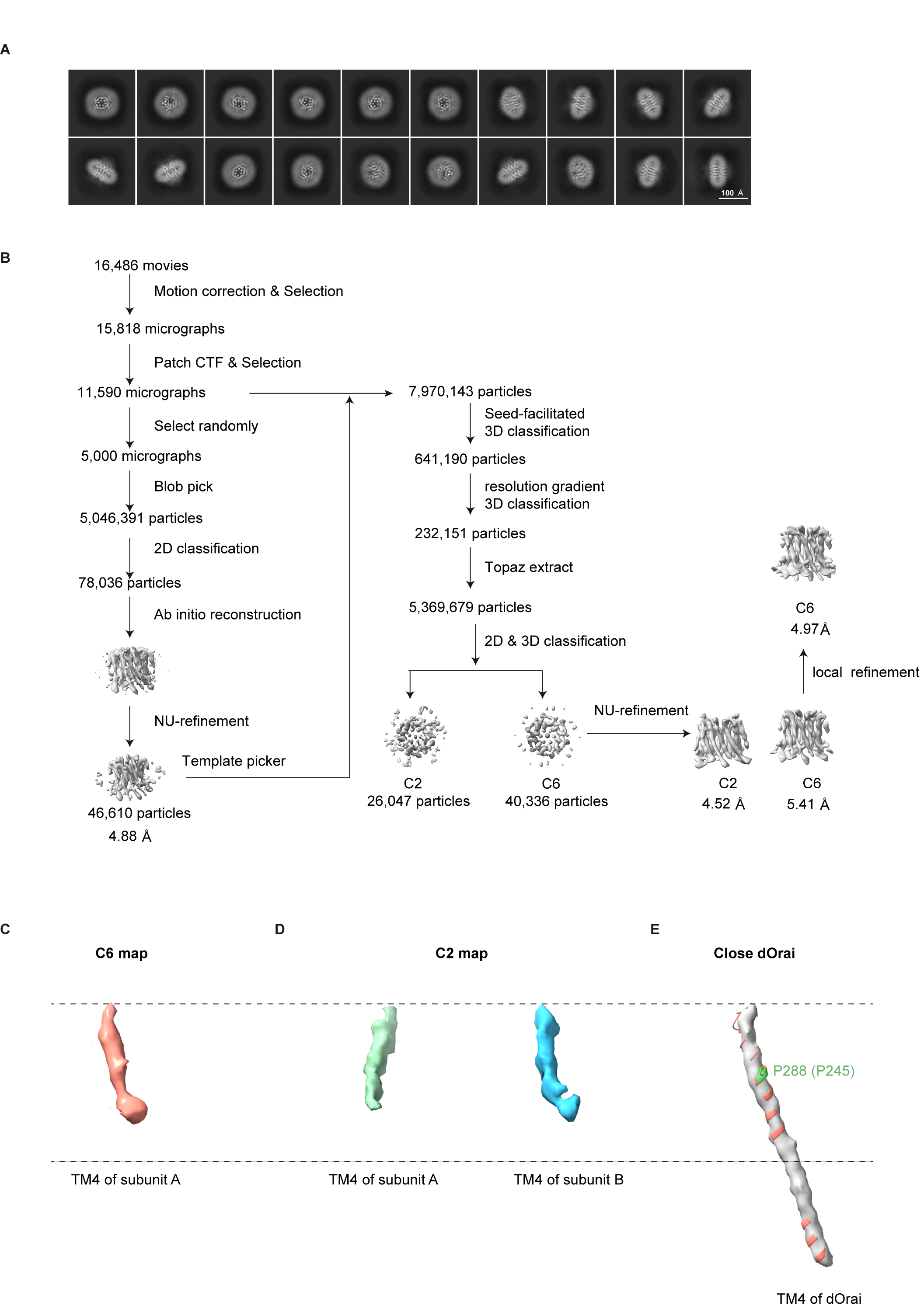

Supplement: S1 Fig — (A) Representative 2D class averages. (B) Cryo-EM data processing workflow in cryoSPARC. (C) EM densities for TM4 helix of hOrai1 with C6 symmetry in Fig 4E are contoured at 7.3 σ. The approximate position of the membrane bilayer is indicated by two dashed lines. (D) EM densities for TM4 helices of hOrai1 with C2 symmetry in Fig 4A are contoured at 9.0 σ. Subunit A and B are represented by green and blue separately. (E) EM densities for the TM4 helix of closed dOrai (PDB: 6BBG), with the position of P288 indicated in green. The corresponding amino acid in human Orai1 is shown in parentheses. (TIF) [file pone.0348440.s001.tif]
