# Supplementary material for: Structural dynamics of the human Orai1 channel revealed by cryo-electron microscopy
Source: PLoS One. 2026 May 11;21(5):e0348440. doi: 10.1371/journal.pone.0348440 (PMC13160330; doi:10.1371/journal.pone.0348440)
Supplement: S1 Table — (DOCX) [file pone.0348440.s002.docx]

**Supplementary Table. 1 | Cryo-EM data collection statistics**

| EMDB ID | C2 symmetry  hOrai1  EMD-66970 | C6 symmetry  hOrai1  EMD- 66969 |  |  |  |
| --- | --- | --- | --- | --- | --- |
| **Data collection and processing** |  |  |  |  |  |
| Magnification | 186,666× | 186,666× |  |  |  |
| Voltage (kV) | 300 | 300 |  |  |  |
| Electron exposure (e^-^/Å^2^) | 50 | 50 |  |  |  |
| Defocus range (μm) | -1.5 to -1.8 | -1.5 to -1.8 |  |  |  |
| Pixel size (Å) | 0.75 | 0.75 |  |  |  |
| Symmetry imposed | *C2* | *C6* |  |  |  |
| Initial particle images (no.) | 5,369,679 | 5,369,679 |  |  |  |
| Final particle images (no.) | 26,047 | 40,336 |  |  |  |
| Map resolution (Å)  FSC threshold | 4.52  0.143 | 4.97  0.143 |  |  |  |
| Map resolution range (Å) | 250-4.52 | 250-4.97 |  |  |  |
|  |  |  |  |  |  |
|  |  |  |  |  |  |
|  |  |  |  |  |  |
|  |  |  |  |  |  |
|  |  |  |  |  |  |
|  |  |  |  |  |  |
|  |  |  |  |  |  |
|  |  |  |  |  |  |
|  |  |  |  |  |  |
|  |  |  |  |  |  |
|  |  |  |  |  |  |
