# Supplementary material for: Structural dynamics of the human Orai1 channel revealed by cryo-electron microscopy
Source: PLoS One. 2026 May 11;21(5):e0348440. doi: 10.1371/journal.pone.0348440 (PMC13160330; doi:10.1371/journal.pone.0348440)
Supplement: S1 File — Uncropped and unadjusted blot and gel images corresponding to Fig 1C and Fig 1D. (PDF) [file pone.0348440.s004.pdf]

All images shown are original, uncropped, and unadjusted. No brightness, contrast, or gamma corrections were applied.

Protein markers used:
PAGE-MASTER Protein Standard Plus (GenScript, Cat. No. MM1397-500)
WB-MASTER Protein Standard (GenScript, Cat. No. M00521)
NativeMark™ Unstained Protein Standard (Thermo Fisher Scientific, Cat. No. LC0725)

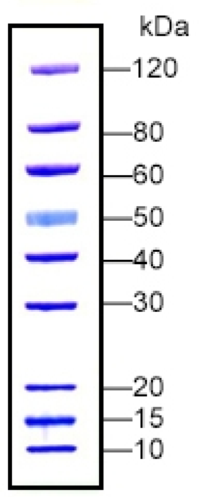

PAGE-MASTER Protein Standard Plus

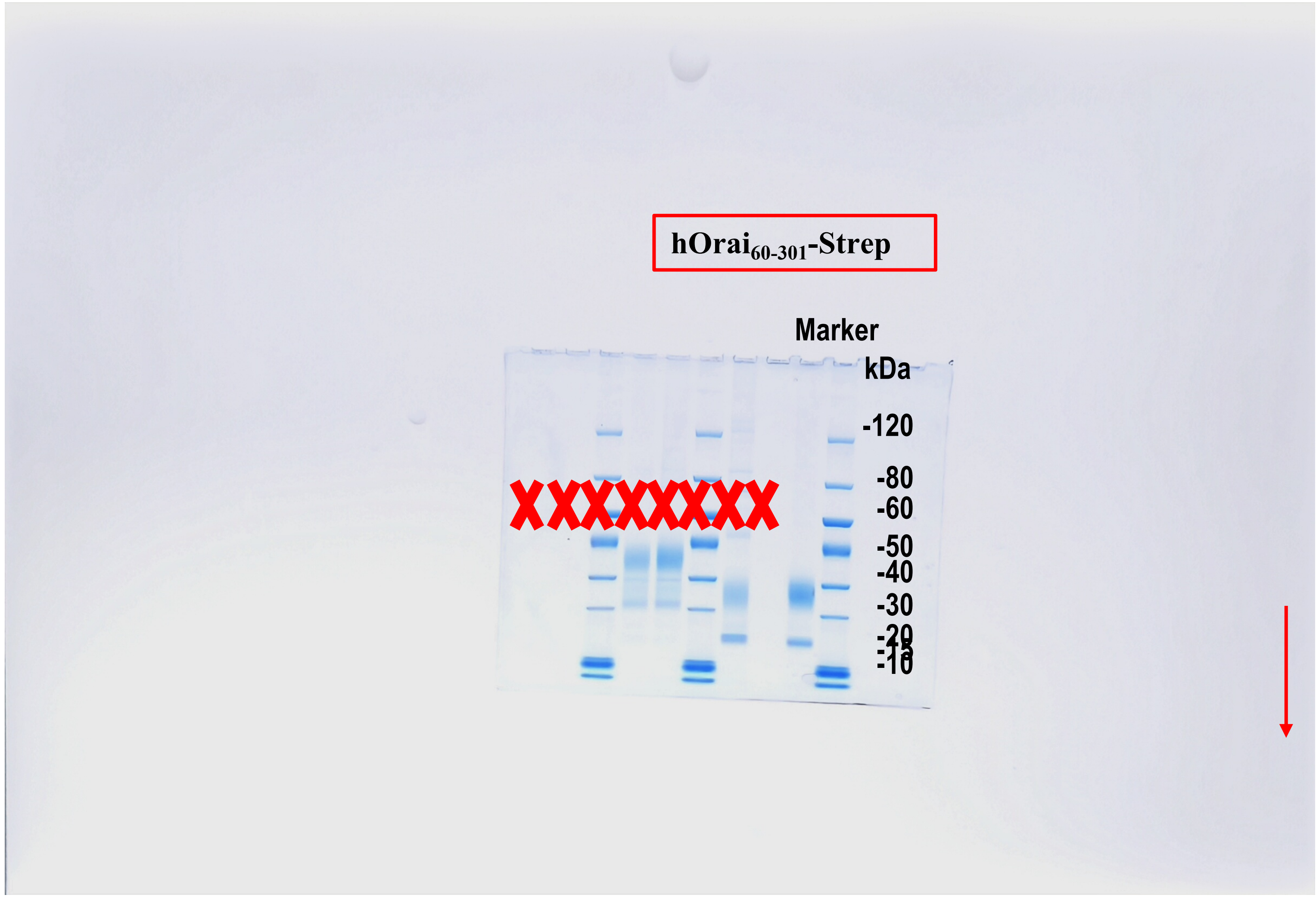

Uncropped SDS PAGE Coomassie-stained gel of hOrai1$_{60–301}$-Strep (Figure 1C, left panel)

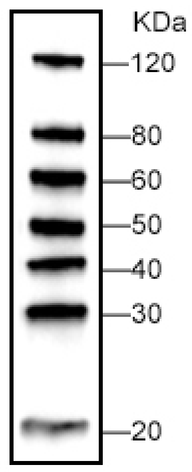

WB-MASTER Protein Standard

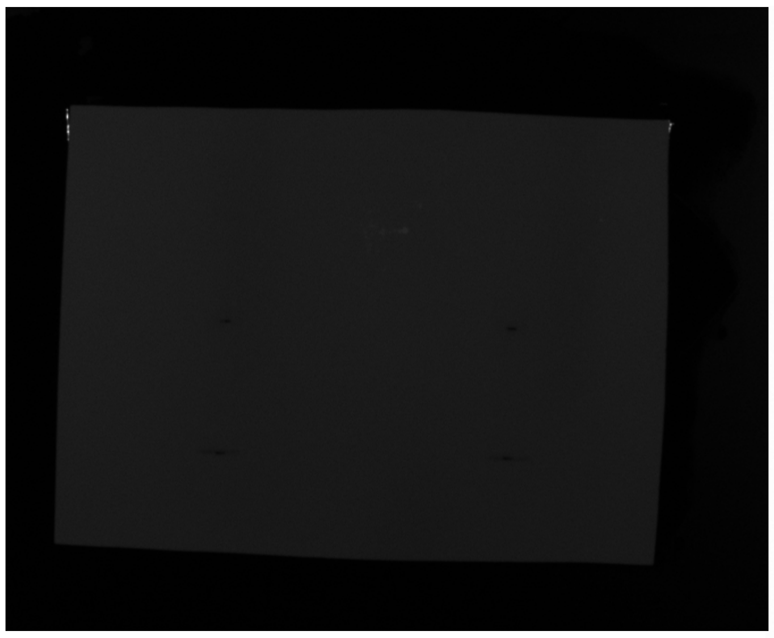

Reference image

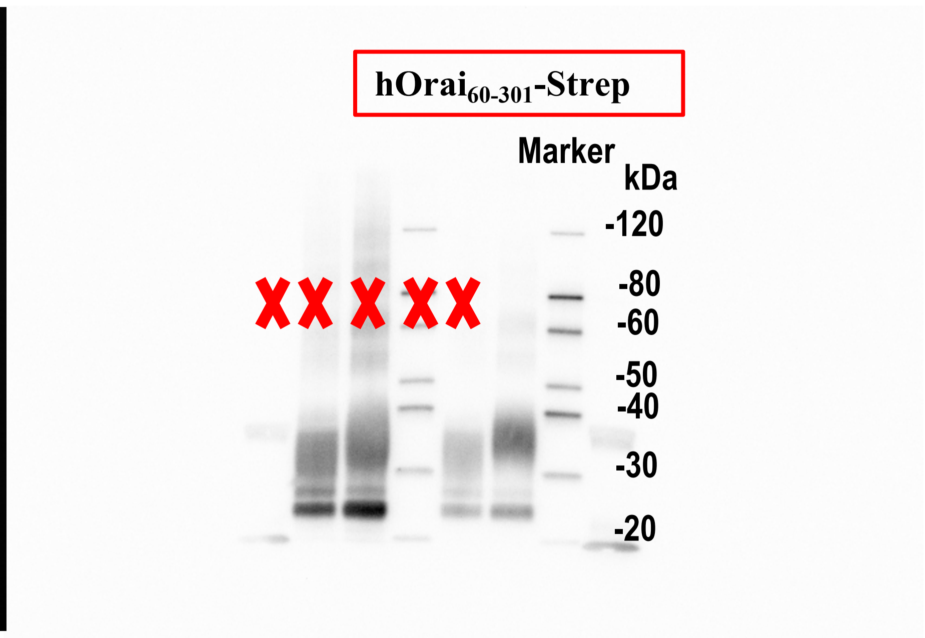

Exposed image

Uncropped SDS-PAGE Western blot of hOrai1$_{60–301}$-Strep (Figure 1C, right panel)

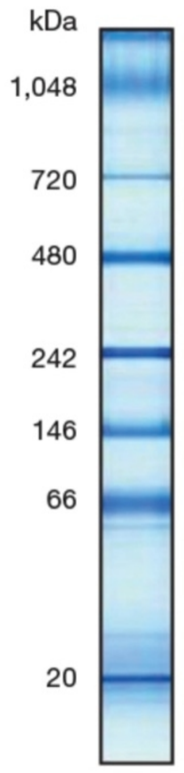

NativeMark™ Unstained Protein Standard

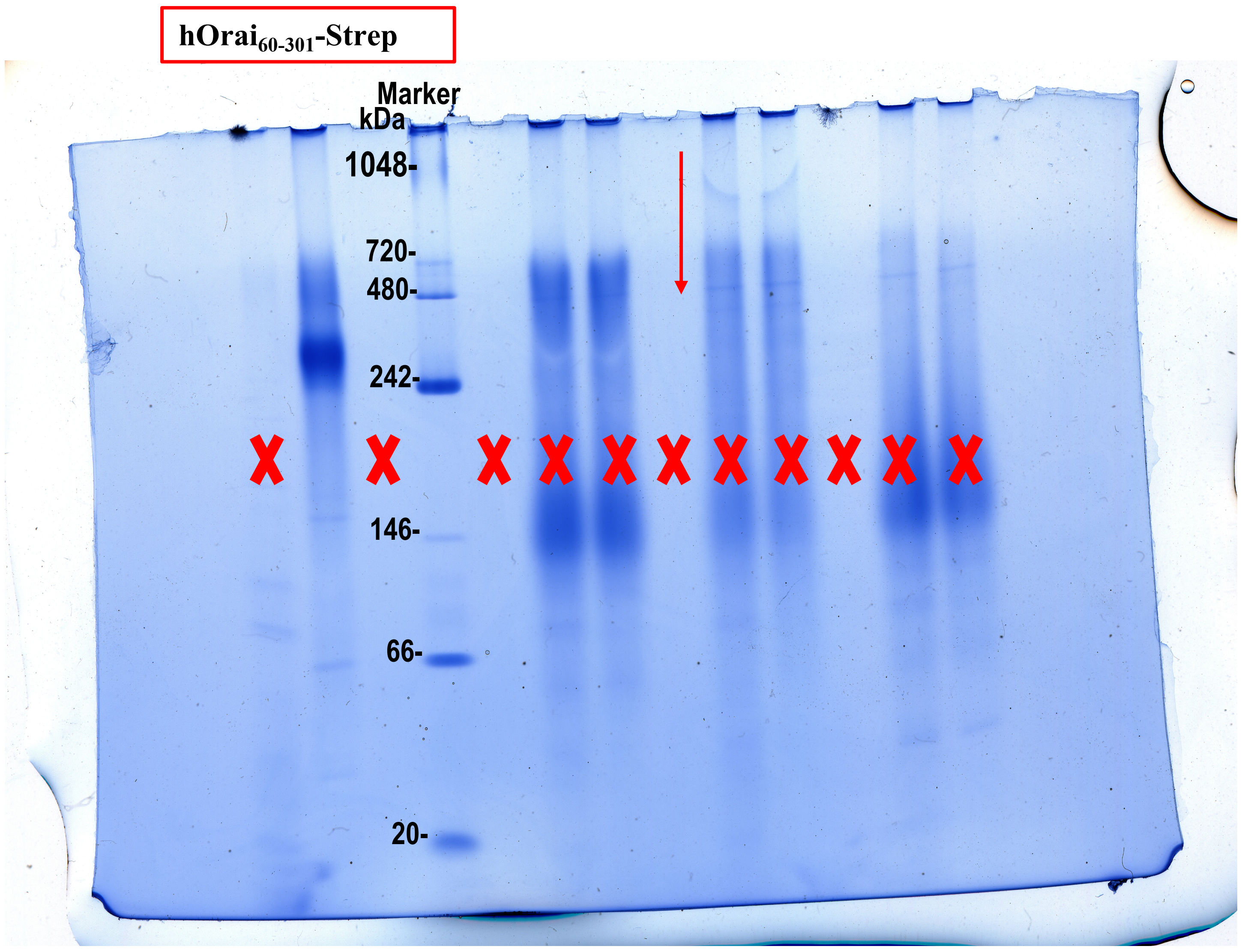

Uncropped Native PAGE Coomassie-stained gel of hOrai1$_{60–301}$-Strep (Figure 1D, left panel)

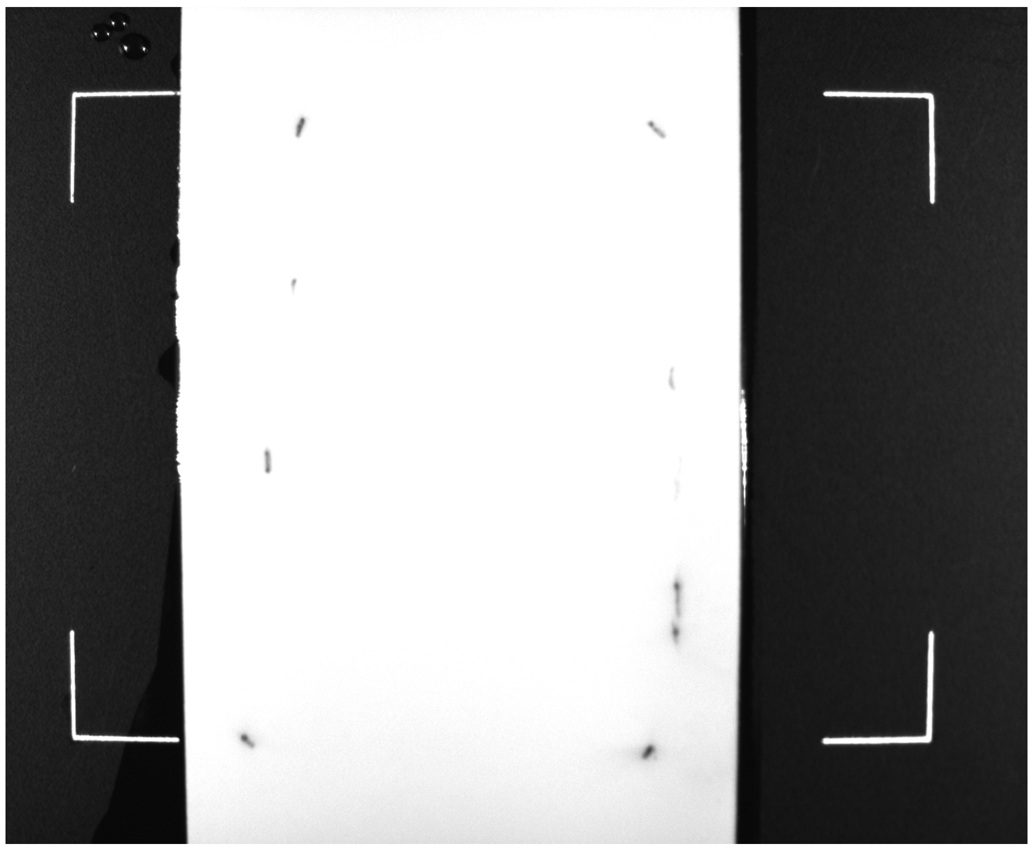

Reference image

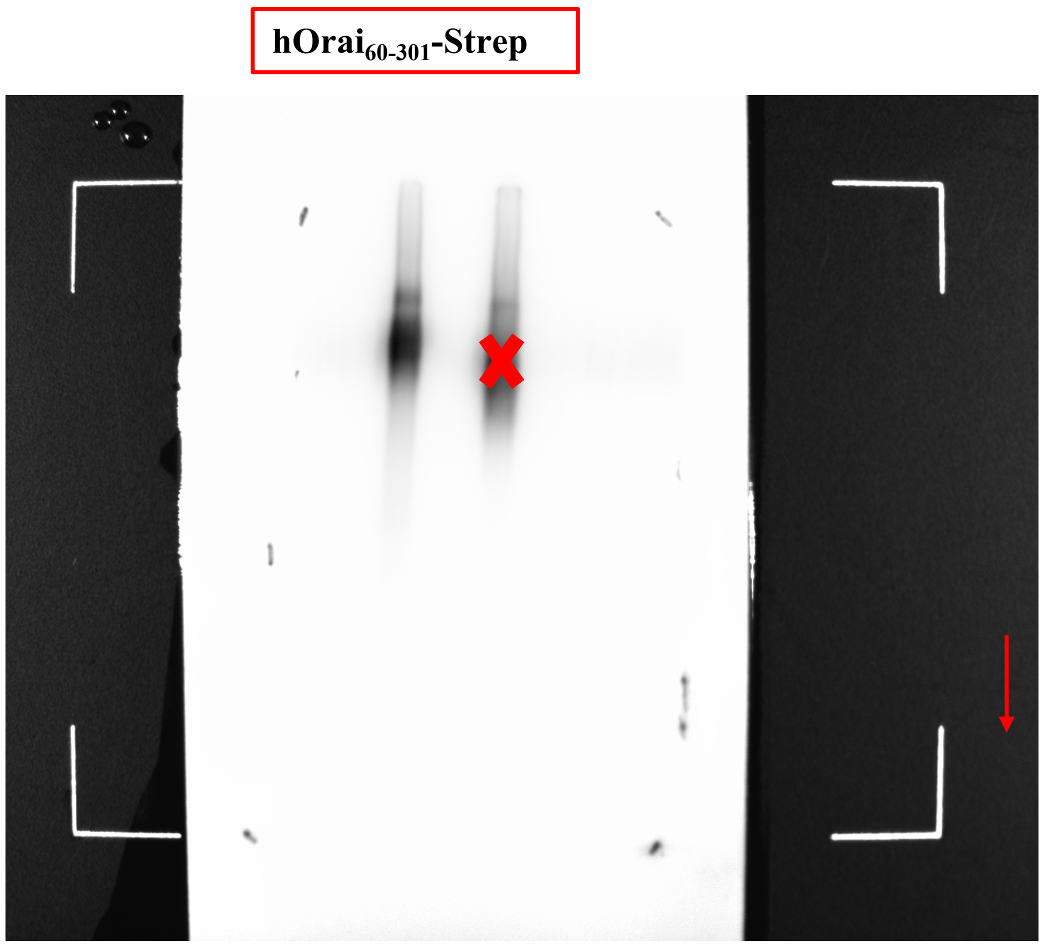

Exposed image

Uncropped Native PAGE Western blot of hOrai1$_{60–301}$-Strep (Figure 1D, right panel)
